# New (Co)poly(hydroxyimide)s Based on 4,4′-Oxydiphthalic Anhydride—Effect of Composition on Properties, Including Gas Transport Ability

**DOI:** 10.3390/ma18102193

**Published:** 2025-05-09

**Authors:** Agnieszka Katarzyna Pająk, Andrzej Jankowski, Ewa Schab-Balcerzak

**Affiliations:** 1 Centre of Polymer and Carbon Materials, Polish Academy of Sciences, 34 M. Curie-Sklodowska Str., 41-819 Zabrze, Poland; ajankowski@cmpw-pan.pl (A.J.); ebalcerzak@cmpw-pan.pl (E.S.-B.); 2 Institute of Chemistry, University of Silesia, 9 Szkolna Str., 40-006 Katowice, Poland

**Keywords:** polyimides, (co)poly(hydroxyimide)s, mechanical properties, gas permeation properties, membrane materials, application materials

## Abstract

This paper presents novel soluble (co)poly(hydroxyimide)s ((co)PIOH) based on 4,4′-oxydiphthalic anhydride (ODPA), 3,3′-dihydroxybenzidine (HAB), and 3,6-diaminodurene (D) with the 3/1, 1/1, and 1/3 HAB/D ratios. This chemical structure of the compounds provides the possibility of their future modification through the thermal rearrangement (polybenzoxazoles) or functionalization via Mitsunobu reaction (azo side-chain polyimides), i.e., obtaining new materials with interesting properties and therefore with expanded applications. Copolymers were characterized via FTIR, NMR, XRD, and GPC methods to confirm their structure, composition, and molar masses. The effect of copolymer composition on the thermal, mechanical, optical, and permeation properties studied for He, O_2_, N_2_, and CO_2_, as well as hydrophobicity, was investigated. They exhibited a large interval between the glass transition temperature and the decomposition temperature, making them promising for the thermoforming technique. Transmittance above 90% was noted in the visible range for all (co)PIOH films deposited on a glass substrate. Young’s modulus of fabricated membranes was in the range of 2.37 to 3.38 GPa. The highest permeability coefficients were recorded for (co)PIOH with a 1:3 HAB-to D-ratio.

## 1. Introduction

The history of aromatic polyimides (PIs) begins in 1908, when Marston Bogert first synthesized the PI from 4-amino phthalic anhydride [1,2]. Then, in the 1960s, the first commercially available polyimide film, Kapton^®^, was produced by DuPont^TM^ (Wilmington, DE, USA) [3,4,5]. Since then, interest in this class of polymers has been growing because it is one of the organic polymers with the highest thermal stability among materials, making it widely used in high-temperature engineering fields [6,7,8,9,10,11,12,13,14,15,16,17]. However, PIs also possess other outstanding properties, such as radiation and chemical resistance, high thermo-oxidative stability, mechanical strength, good film-forming ability, and low dielectric constant and accessible production pathways. They have been employed as adhesives, matrices of composites, fibers, films, and foams in high-temperature engineering of microelectronics, microelectromechanical systems, photoelectronics, alignment layers in liquid crystal displays, and aerospace industries, as well as membranes for gas separation [18,19,20,21,22,23,24,25,26,27,28,29,30,31,32,33]. In addition to classical applications, polyimides are being explored as very promising active materials for optoelectronics as fluorescent species and the new class of redox-active and electrochromic polymers. Considering the importance of “green chemistry” and sustainable development, it is predicted that biomass-based polyimide may displace conventional petroleum-based PIs [34]. One of the most remarkable features of PI is its flexibility, which allows it to change its properties as desired by changing its molecular structure. Thus, significant effort has been made to improve their properties via chemical modification of the chain backbones and higher-order structure control. A convenient method of modification is copolymerization, allowing us to obtain materials with the desired properties [35,36,37,38,39].

Among the extensive group of PIs, noteworthy are polyimides functionalized with hydroxyl groups—poly(hydroxyimide)s. The presence of hydroxyl groups gives the possibility for modification based on the introduction of azo chromophores to obtain photoactive polymers. As a consequence of polarized light’s exposure to the isotropic azo polymers, we can obtain the anisotropic materials (the phenomenon of birefringence and photoinduced dichroism, and the formation of diffraction gratings). Materials with such properties can be used as photoactive systems; photoswitchable binary switches (remote switching by light); and photomechanical triggers for mechanical, electrical, and optical mechanisms [40,41,42,43]. Moreover, by irradiating with light of an appropriate wavelength, for the polyimide membrane with azo fragments incorporated into the main chain of the polymer backbone, we are able to take control of the gas separation, which is a promising phenomenon for future applications of these materials. By attaching azo groups as side-chain elements to the polymer backbone, higher gas permeability can also be achieved for these compounds than for their analogues with azo dyes dispersed in the polymer matrix [43]. Furthermore, the next possible modification of poly(hydroxyimide)s is thermal rearrangement, leading to the formation of rigid polybenzoxazole (PBO) rings being desired for the fabrication of membranes for highly efficient natural gas separation and purification. PBOs are characterized by good thermal stability and solvent resistance. Thermal rearrangement results in chain stiffening and pore reconfiguration of the tested material, which increases the fractional free volume and influences the uniform distribution of free volume elements. Therefore, membranes of PBOs exhibit the high gas separation efficiency and also good plasticization resistance [44,45,46,47,48].

In this study, the novelty aspect was obtaining materials possessing a new chemical structure providing of their further modification’s the possibility by functionalization of hydroxyl groups. We synthesized the three (co)poly(hydroxyimide)s ((co)PIOHs) using a 4,4′-oxydiphthalic anhydride (ODPA) and a mixture of 3,3′-dihydroxybenzidine (HAB) with 3,6-diaminodurene (D) in three different molar ratios (3/1, 1/1, and 1/3 HAB/D) and comprehensively characterized them. Thus, this research concerns their thermal (TGA and DSC), hydrophobicity (the water contact angle), and optical (UV-Vis, ultraviolet, and visible ranges of the spectrum) measurements in the solid state as a thin film on glass, as well as their mechanical and gas transport properties (permeation behavior of N_2_, O_2_, He, and CO_2_). For comparison, the obtained results for analogous polyimides are also presented in this paper. The polymer ODPA-D has already been described in a publication by our research group [49]. Moreover, comparisons of the selectivity and permeability with some common membranes of commercial materials (Matrimid^®^ 5218 (Vantico Inc., Luxembourg), Ultem^®^ 1000 (GE Plastics, Mount Vernon, IN, USA) and Kapton^®^ (DuPont^TM^, Wilmington, DE, USA)) are given.

## 2. Experimental Section

### 2.1. Materials

3,6-diaminodurene (D), 3,3′-dihydroxybenzidine (HAB), 4,4′-oxydiphthalic anhydride (ODPA), anhydrous N,N-Dimethylformamide (DMF), anhydrous N-methyl-2-pyrrolidinone (NMP), and o-dichlorobenzene (ODB) were purchased from Sigma-Aldrich (Merck, Steinheim, Germany). Chloroform, dimethyl sulfoxide (DMSO), methanol, and tetrahydrofuran (THF) were received from Chempur (Piekary Śląskie, Poland). Nitrogen, helium, carbon dioxide (purity 99.998%), and oxygen (purity 99.95%) were purchased from Messer Poland S.A. (Chorzów, Poland).

### 2.2. Synthesis of Polyimides and (Co)poly(hydroxyimide)s

Polyimides (ODPA-HAB and ODPA-D) were obtained via a one-step polycondensation reaction of the dianhydride (ODPA) with an equimolar amount of diamine (HAB or D) in a mixture of N-methyl-2-pyrrolidinone and o-dichlorobenzene (4:1; *v*:*v*), according to the described procedure [49,50]. The reaction mixture (20% monomer concentration) was vigorously stirred for 2–3 h, gradually increasing the temperature to 180 °C. The imidization reaction was carried out in solution at 180 °C for 3.5 h. After the reaction was completed and the reaction mixture was cooled to room temperature, the polyimide was isolated from the solution by precipitation with a non-solvent. The crude product was purified by dissolving in DMF and reprecipitating. The polyimides were extracted with methanol in the Soxhlet apparatus for 2–3 days and dried in a vacuum dryer by gradually increasing the temperature to 150 °C.

(Co)poly(hydroxyimide)s (ODPA-HAB(D)s) were also obtained by a one-step polycondensation reaction, using mixtures of dianhydride (ODPA) and diamines (HAB or D) in different molar ratios (3:1, 1:1, and 1:3) in a mixture of NMP and ODB (4:1; *v*:*v*). The reaction mixture was stirred and heated under similar conditions to those used in the case of the obtainment of the abovementioned polyimides. The polymer solution was cooled to room temperature, and then the polymer was precipitated in methanol or distilled water. The crude product was purified by dissolving it in DMF and reprecipitating it in methanol or distilled water. The (co)poly(hydroxyimide)s were extracted with methanol in the Soxhlet apparatus for 2–3 days and dried in a vacuum by gradually raising the temperature to 150 °C [49,50].

ODPA-HAB: ATR FTIR (cm^−1^): 3363 (–OH), 1776, 1704 (imide C=O), 1375 (imide –C–N–), 1231 (–C–O–C–), 744 (imide –C–N–). Yield: 99%.

ODPA-D: ATR FTIR (cm^−1^): 2963 (–CH_3_), 1779, 1713 (imide C=O), 1350 (imide –C–N–), 1225 (–C–O–C–), 747 (imide –C–N–). Yield: 97%.

(co)PIOH-1 (ODPA-HAB(D) (3:1)): ^1^H NMR (600 MHz, DMSO-d_6_, δ, ppm): 10.10 (s, 2H,–OH), 8.22–7.95 (m, 4H, ArH), 7.89–7.51 (m, 8H, ArH), 7.40–7.35 (m, 2H, ArH), 7.28–7.10 (m, 4H, ArH), 2.06 (s, 12H, –CH_3_). ATR FTIR (cm^−1^): 3382 (–OH), 2929 (–CH_3_), 1776, 1705 (imide C=O), 1359 (imide –C–N–), 1224 (–C–O–C–), 745 (imide –C–N–). Yield: 99%.

(co)PIOH-2 (ODPA-HAB(D) (1:1)): ^1^H NMR (600 MHz, DMSO-d_6_, δ, ppm): 10.09 (s, 2H,–OH), 8.34–7.92 (m, 4H, ArH), 7.89–7.45 (m, 8H, ArH), 7.39–7.35 (m, 2H, ArH), 7.28–7.13 (m, 4H, ArH), 2.06 (s, 12H, –CH_3_). ATR FTIR (cm^−1^): 3373 (–OH), 2929 (–CH_3_), 1776, 1709 (imide C=O), 1355 (imide –C–N–), 1223 (–C–O–C–), 745 (imide –C–N–). Yield: 98%.

(co)PIOH-3 (ODPA-HAB(D) (1:3)): ^1^H NMR (600 MHz, DMSO-d_6_, δ, ppm): 10.13 (s, 2H,–OH), 8.20–8.02 (m, 4H, ArH), 7.84–7.60 (m, 8H, ArH), 7.38–7.36 (m, 2H, ArH), 7.25–7.15 (m, 4H, ArH), 2.06 (s, 12H, –CH_3_). ATR FTIR (cm^−1^): 3386 (–OH), 2925 (–CH_3_), 1776, 1719 (imide C=O), 1356 (imide –C–N–), 1225 (–C–O–C–), 746 (imide –C–N–). Yield: 96%.

### 2.3. Membranes Formation

Membranes were prepared from the polyimides’ homogenous solutions in anhydrous DMF (80 g/dm^3^). The formed samples were dried at 50 °C for 48 h, and next dried at 80 °C for 18 h in the vacuum oven. Next, the temperature was raised slowly to 150 °C, and the membranes were heated at this temperature for an additional 18 h. They were additionally dried in a full vacuum overnight (argon atmosphere). The thickness of the prepared samples was in the range of 35–49 µm.

### 2.4. Characterization Methods

Nuclear magnetic resonance spectra were recorded on an Avance II UltraShield Q3 Plus Bruker MT 600 MHz (Bruker Corporation, Billerica, MA, USA). Infrared spectra of the polyimide films were recorded on a Nicolet 6700 FTIR apparatus (Thermo Fisher Scientific, Waltham, MA, USA). The prepared samples were analyzed using the attenuated total internal reflection method of infrared radiation (the ATR attachment with a diamond crystal). The X-ray diffraction patterns of polyimide membranes on a wide-angle HZG-4 diffractometer (Carl Zeiss, Jena, Germany) working in the typical Bragg geometry and using CuKα radiation were recorded. The gel permeation chromatography (GPC, gel chromatograph with MALLS and refractometric detector system) was used to determine the molecular weights (M_n_ and M_w_) and dispersity (M_w_/M_n_). Measurements were performed with polystyrene standards and DMF as eluent. The water contact angle of the membrane surface was determined using a CAM 101 optical goniometer (KSV Instruments Ltd., Helsinki, Finland) at room temperature using the static sessile drop method. Thermal studies were performed using a TA-DSC 2010 TA Instruments (Newcastle, DE, USA) with a heating/cooling rate of 20 °C·min^−1^ under nitrogen, a TGA/DSC 1 STARe system SW 9.30 software Mettler Toledo (Mettler Toledo, Greifensee, Switzerland) with a heating rate of 10 °C·min^−1^ in a constant stream of nitrogen, and a temperature range from 25 °C to 800 °C. The transmittance spectra in the UV-Vis range of (co)PIOHs’ films (NMP solution c = 0.004 g/cm^3^) were performed using Jasco V-750 (Jasco Inc., Tokyo, Japan). The mechanical properties were performed with an Instron Model 4204 tensile tester (Instron, Norwood, MA, USA) with a 20 mm·min^−1^ tensile speed at 25 °C (the samples were 10 mm wide and 30 mm long, with three samples for each material). The tensile strength and elongation at break were directly determined from the plots. Young’s modulus was estimated from the stress–strain plot for the initial linear region. The tests of polyimides’ density were carried out using the buoyancy method based on Archimedes’ principle. Measurements were taken by weighing prepared films in air and immersed in water or isooctane. The densities of polyimides, *ρ*, were calculated from the following equation:

(1) $$\rho =\frac{m_{a}}{m_{a}-m_{l}}\rho_{l}$$

where *m_a_* and *m_l_* are the samples’ masses measured in air and in liquid, respectively; and *ρ_l_* is the liquid density. Gas transport properties were examined by using a constant volume apparatus. The membranes were degassed in an apparatus cell for at least 10 h (the downstream and upstream sides) at 30 °C. The permeation of pure N_2_, O_2_, He, and CO_2_ was measured at 30 °C under a 6 bar pressure (upstream side). The gas permeability was calculated from the following formula:

(2) $$P=10^{-10}\frac{V_{d}l}{{RTAp}_{2}}\left[{\left(\frac{{dp}_{1}}{dt}\right)}_{ss}-{\left(\frac{{dp}_{1}}{dt}\right)}_{leak}\right]$$

where *P* is the gas permeability (Barrer); *V_d_* is the downstream volume (cm^3^); *l* is the thickness of the membrane (cm); *R* is the gas constant (cm Hg·cm^3^·cm^−3^ (STP) K^−1^); *T* is the absolute temperature (K); *A* is the effective area of the membrane (cm^2^); *p*_2_ and *p*_1_ are the absolute upstream pressure (cm Hg); and $\frac{{dp}_{1}}{dt}$ is the change in absolute downstream pressure observed during the experiment for steady-state and for leak conditions (indexes “*ss*” and “*leak*”, respectively). The values of the gas permeability coefficient, *P*, determined by the presented method were burdened with an error of less than 10%. The “ideal selectivity”, *α_AB_*, of the membrane for two gasses, *A* and *B*, was calculated as a ratio of the pure gas permeabilities, according to the following expression:

(3) $$\alpha_{AB}=\frac{P_{A}}{P_{B}}$$

The fractional free volume (*FFV*) was estimated from the following equation:

(4) $$FFV=\frac{V-V_{0}}{V}$$

where *V* is the molar volume at temperature, T (determined experimentally as the reciprocal of the measured polymer density); and *V*_0_ is the molar volume occupied by the polymer chains at 0 K. The *V*_0_ is estimated using the van der Waals volume (*V_W_*) from the following formula:

(5) $$V_{0}=1.3V_{W}$$

## 3. Result and Discussion

### 3.1. The (Co)polyimides Characterization

**Figure 1:**
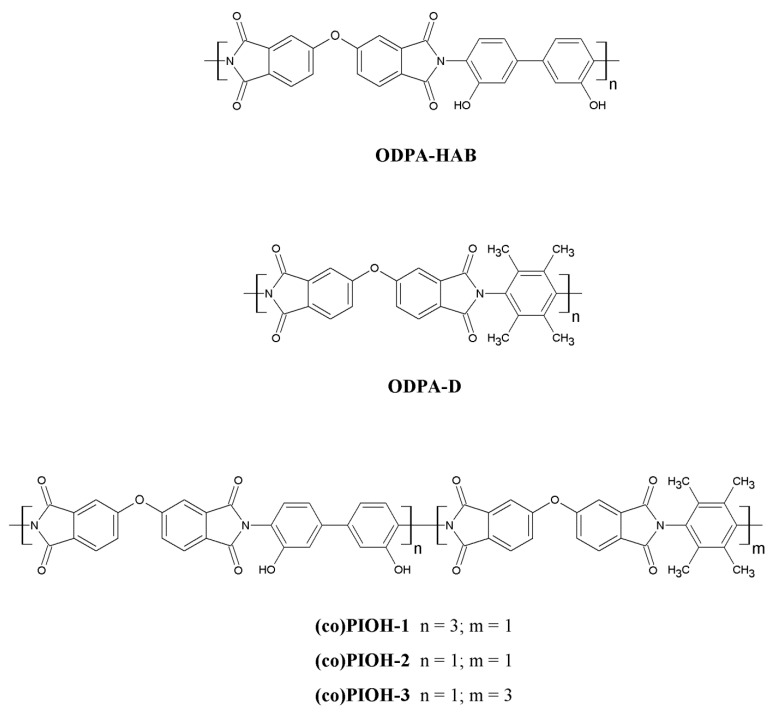
Chemical structures of the obtained (co)poly(hydroxyimide)s ((co)PIOHs).

**Figure 2:**
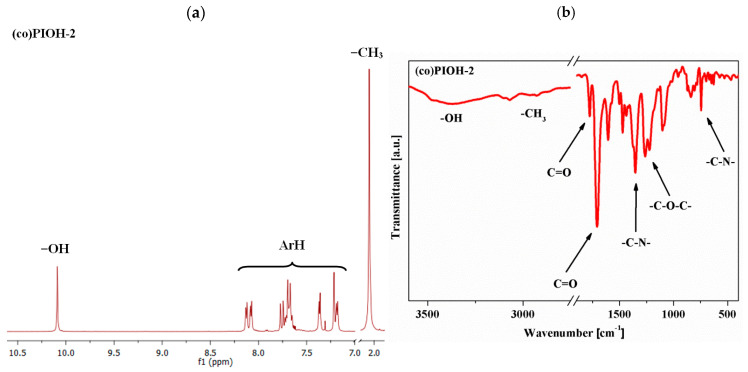
The selected (**a**) ^1^H NMR and (**b**) FTIR spectra of the (co)PIOH-2.

The chemical structures of (co)poly(hydroxyimide)s are presented in Figure 1. The ^1^H NMR and ATR FTIR were used to prove the chemical structures of the synthesized (co)PIOHs. The selected ^1^H NMR and FTIR spectra are shown in Figure 2.

In the ^1^H NMR spectra, the characteristic signal of the molecule group’s –OH was in the range of 10.13–10.09 ppm. The protons of the compound’s aromatic part were recorded in the range of 8.34–7.10 ppm, and for the groups of –CH_3_, it can be seen at 2.06 ppm (Figure 2a). In these spectra, signals above 10.50 ppm were not seen, and in ATR FTIR spectra, the lack of amide group’s absorption bands at 1650 cm^−1^ was confirmed, indicating that the complete conversion of poly(amic acid) to polyimide had occurred. Moreover, the characteristic imide absorption bands, namely symmetric C=O stretch at 1776 cm^−1^, asymmetric C=O stretch at 1719–1705 cm^−1^, –C–N– stretch at 1359–1355 cm^−1^, and –C–N– ring deformation at 746–745 cm^−1^, were also detected. The FTIR bands at 3386–3373 cm^−1^ (–OH) and at 2929–2925 cm^−1^ (–CH_3_) were also observed (Figure 2b).

**Figure 3:**
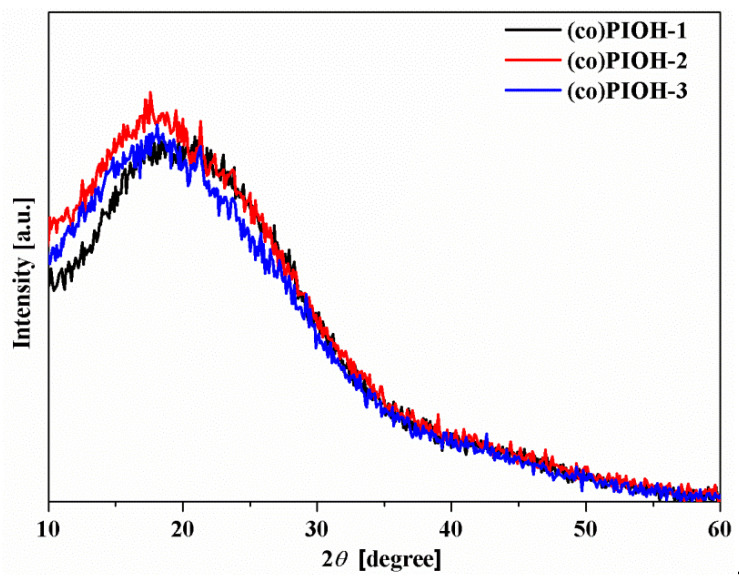
X-ray diffraction patterns of the examined (co)PIOHs.

The structures of (co)PIOHs were verified by the wide-angle X-ray diffraction measurements (WAXD). The broad halo (the peak in the range of 16–21°) without any crystalline peaks indicated that all the investigated (co)PIOHs were amorphous (Figure 3).

Then, the d-spacing values from the position of the diffraction maximum were estimated. The higher content of the D diamine in the molecule, the higher d-spacing value, indicating worse packing of the polymer chains (Table 1).

The increasing share of the D unit in the molecule caused a slight decrease in the density of the tested compounds. Density measurements of the (co)PIOH also confirmed increased steric hindrance in the packing of the polymer chain (volume of the D segment compared to the HAB-containing segment) [51]. Furthermore, the fractional free volumes (FFVs) of polyimide materials were calculated using the determined densities and the Bondi contribution group method [52]. The presence of –CH_3_ groups in the benzene ring of D units increased the FFV parameter for the (co)PIOHs (Table 1).

**Figure 4:**
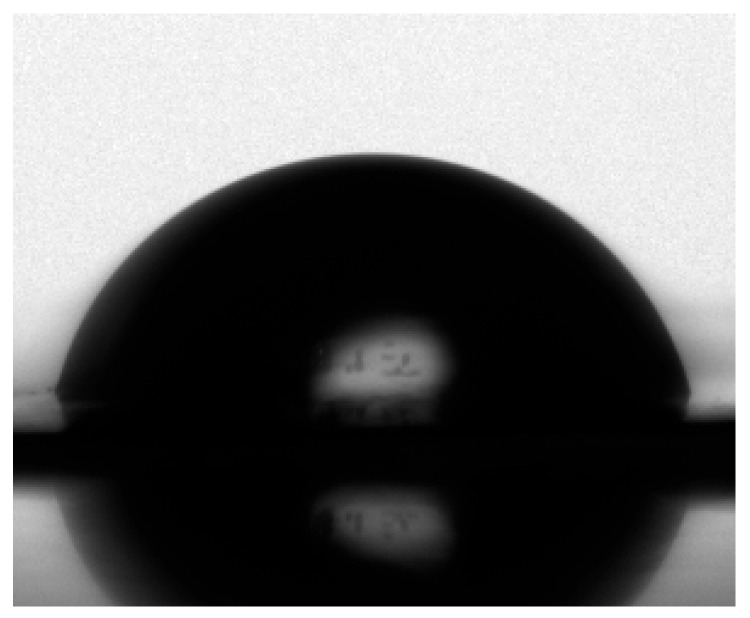
The water contact angle of the (co)PIOH-2.

The tested (co)poly(hydroxyimide)s exhibited good solubility in NMP, DMF, and DMSO at room temperature, and in THF and CHCl_3_, they were also partially soluble upon heating. Additionally, the water contact angle of the prepared (co)PIOHs membranes was determined. The contact angle was 66° for the (co)PIOH-1, 80° (co)PIOH-2 (Figure 4), and 67° (co)PIOH-3. It was observed that the contact angle is lower for compounds with higher amounts of ODPA ((co)PIOH-1 and (co)PIOH-3). This was most likely due to the hydrogen bond’s formation between oxygen (from an ether linkage with ODPA) and water [53].

The number (M_n_) and weight (M_w_) average molar masses of (co)PIOHs were evaluated by GPC analysis in DMF solvent using polystyrene standards. The obtained results are listed in Table 1. The obtained M_n_ masses of the examined (co)PIOHs were in the range of 34,000–89,000 g/mol, while the M_w_ masses were in the range of 80,000–209,000 g/mol. The dispersity (M_w_/M_n_) of (co)PIOHs was in the range of 2.3–2.6 (Table 1), which is typical for polymers from polycondensation processes [54]. The properties of the synthesized (co)poly(hydroxyimide)s allowed for the preparation of flexible and good-quality membranes.

### 3.2. Thermal Properties

The thermogravimetric analysis (TGA) and the differential scanning calorimetry (DSC) were evaluated to examine the thermal properties of the (co)PIOHs. The results are collected in Table 2.

The synthesized compounds exhibited a temperature of 5% weight loss (T_5%_) above 370 °C during heating in a nitrogen atmosphere. The highest T_5%_ (386 °C) was recorded for the (co)PIOH-2, i.e., for ODPA-HAB(D) 1:1. Furthermore, the residual weight for all (co)PIOHs was about 60% at 800 °C. As described in the literature and also reported in our research group, for (co)poly(hydroxyimide)s, it is usually noted that there are two or three degradation steps [42,43,55,56,57,58]. In the case of the analyzed (co)PIOHs, we observed two major thermal degradation regions. The first region was in the range of 407–432 °C, and it was assigned with decarboxylation (carbon dioxide was emitted). The hydroxyl groups of polyimides can undergo molecular thermal conversion to polybenzoxazoles under an inert atmosphere. The temperatures at which this transformation occurs are much higher than the T_g_ of the (co)poly(hydroxyimide)s. The second region (508–521 °C) was shown the thermal decomposition of the aromatic polyimide segments (Table 2). Similar behaviour was observed in previous publications of our research group for structurally comparable compounds (among other things, different connections between the imide rings in both parts of the copolyimide) [42,43,55,57]. Comparing the obtained results of the first temperature of the maximum decomposition (T_max_) for (co)PIOHs, it was noticed that increasing the amount of D ((co)PIOH-3) in the molecule resulted in the first T_max_’s increase by more than 20 °C and the second T_max_’s decrease of more than 10 °C.

**Figure 5:**
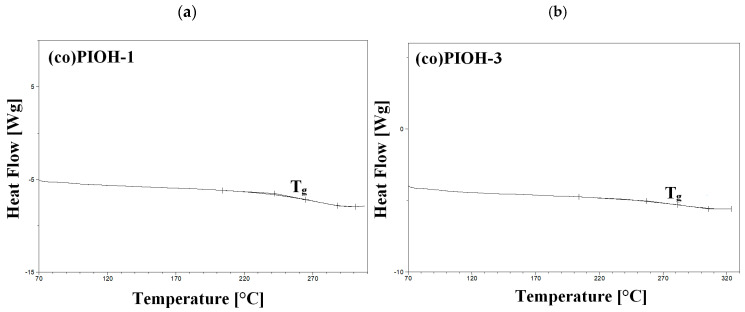
The DSC thermograms of the (**a**) (co)PIOH-1 and (**b**) (co)PIOH-3 (registered in II heating scan and exo up).

In the DSC thermograms, no melting peaks were registered, only (co)PIOHs’ glass transition, which confirms that all the studied (co)poly(hydroxyimide)s were amorphous. The glass transition temperature (T_g_) was registered during the second heating scan in the range of 257–281 °C (Table 2). The highest T_g_ (281 °C) was recorded for the (co)PIOH-3, which was probably caused by the stiffening of the polymer chain due to the hindered rotation of the benzene ring with the –CH_3_ groups (Figure 5b). Moreover, comparing the T_g_ of the (co)PIOH-1 (265 °C) and (co)PIOH-2 (257 °C), a decrease in T_g_ with a decreasing amount of –OH groups was observed (reduced intermolecular attraction forces in hydrogen bond formation) (Table 2 and Figure 5a) [55].

### 3.3. Optical and Mechanical Properties

**Figure 6:**
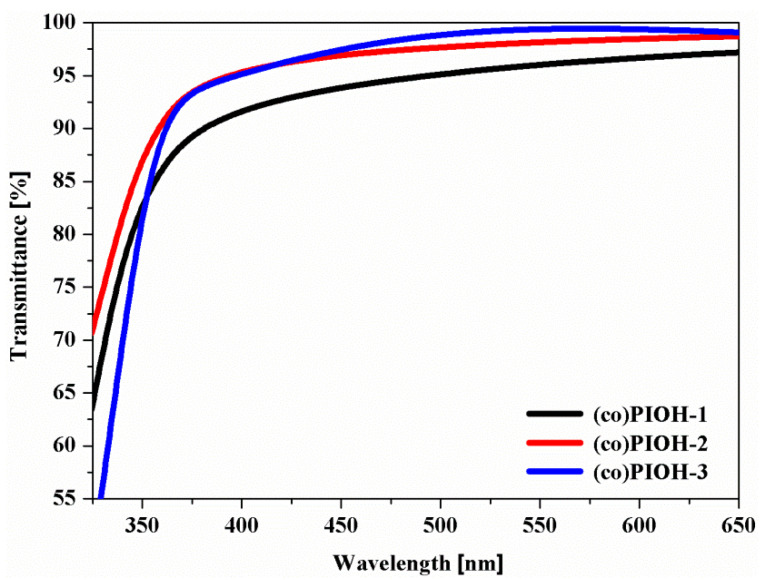
UV-Vis transmittance spectra of the (co)PIOHs films.

In the Vis range transmittance above 90% was noted for the recorded UV-Vis spectra of (co)PIOHs’ films, whereas in the UV range, a decrease in transmittance was observed (Figure 6).

The tested films exhibited good visible transparence. In the Vis range, the most incident radiation was transmitted by the (co)PIOH-3 and the least by the (co)PIOH-1 (with the increasing of the HAB contents in the polymer).

The mechanical properties of the (co)PIOHs membranes were investigated. Young’s modulus (E), tensile strength (R_m_), and elongation at break (A) are gathered in Table 3.

The tensile strength, elastic modulus, and elongation at break ranged from 101.1 to 184.1 MPa, from 2.37 to 3.38 GPa, and from 2.3 to 2.7%, respectively. The highest mechanical parameters were recorded for the (co)PIOH-1 compound, and the lowest for the (co)PIOH-3 (Table 3). In the ODPA-HAB(D) system, as the molar ratio changes, the presence of more HAB units contributed to improving the mechanical properties of the examined materials. Meanwhile, the presence of more D units worsened the tested properties, which was most likely due to the stiffening effect of the (co)polyimide’s chains. With the increase in T_g_, a decrease in both the tensile strength and elongation at the break of these compounds was observed [57].

### 3.4. Gas Transport Properties

The permeation coefficients of pure gases for the examined membranes were determined based on the results of transport measurements using a constant-volume system. The gas transport investigations of prepared (co)PIOHs’ membranes were performed under an upstream pressure of 6 bar and at 30 °C. The permeability coefficients and the ideal selectivity (α) to N_2_, O_2_, He, and CO_2_ are collected in Table 4.

**Figure 7:**
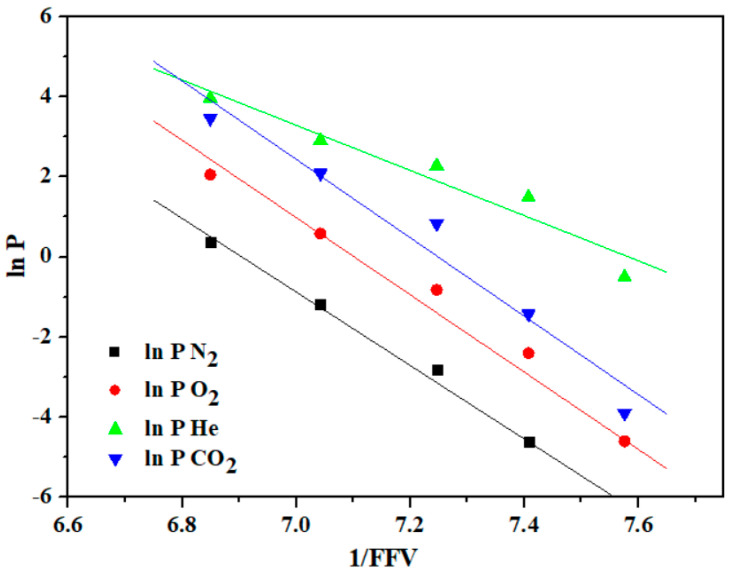
The ln gas permeability with 1/FFV for the tested (co)PIOH membranes.

The permeation behavior of He (2.60 Å) > CO_2_ (3.30 Å) > O_2_ (3.46 Å) > N_2_ (3.64 Å) of all tested membranes was consistent with the order of increasing gas kinetic diameters that is characteristic of glassy polymers [49,58]. While analyzing the above-mentioned properties of the tested (co)PIOHs, the influence of the copolyimides’ chemical structure on their transport properties was noted. An increase in the gas permeation coefficient was observed with the increasing content of D units in the (co)PIOH chain. The highest permeability coefficients were recorded for the (co)PIOH-3 (three units of D) (Table 4). As already mentioned, –CH_3_ groups could hinder the rotation of polymer segments (reducing chain flexibility), resulting in the highest T_g_ for the (co)PIOH-3 among the tested compounds and the best permeation properties for this compound. Furthermore, the increase in the estimated tested compounds’ d-spacing with the increase in permeation indicated that the (co)PIOH-3 had a more open structure than the (co)PIOH-1 (there may be disruptions in packing) [59,60]. The assumed low gas permeability for ODPA-HAB was also confirmed. Moreover, the ideal selectivity O_2_/N_2_ and CO_2_/N_2_ were calculated. The highest α O_2_/N_2_ was obtained for the compound (co)PIOH-1, and the highest α CO_2_/N_2_ for the (co)PIOH-2 (Table 4). The increase in the content of ODPA-D units in (co)PIOH causes a decrease in the value of α O_2_/N_2_. Figure 7 shows the correlation between ln P (ln permeability) and the inverse of the fractional free volume.

The calculated coefficients of determination (r^2^) ranged from 0.912 to 0.991. For the investigated membranes, good linear relationships were obtained between variables. The decreasing nature of these functions was in accordance with the Cohen–Turnbull model [61].

**Figure 8:**
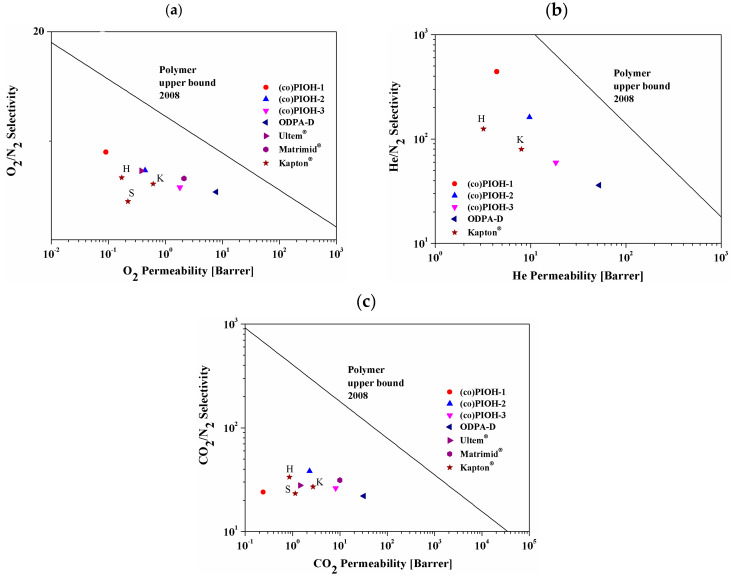
Permeability/selectivity plots for studied (co)PIOHs and commercial materials with respect to the following gas pairs: (**a**) O_2_/N_2_, (**b**) He/N_2_, and (**c**) CO_2_/N_2_ [62,63,64,65,66,67].

The values of permeability and selectivity for the O_2_/N_2_, He/N_2_, and CO_2_/N_2_ gas pairs are presented for tested membranes of (co)PIOHs and commercial materials (Matrimid^®^, Ultem^®^ and Kapton^®^) in the form of Robeson’s diagram in Figure 8. Moreover, the permeation coefficients and the ideal selectivity for commercial materials are gathered in Table 5 [62,63,64,65,66,67].

All the investigated compounds exhibited a performance located below the polymer 2008 upper bound. Comparing the combination of selectivity and permeability in the presented Robeson’s diagrams, we see that the obtained values for (co)PIOHs were not significantly different from those of commercial polymers for O_2_/N_2_ and CO_2_/N_2_. For the He/N_2_ separation, the transport properties of (co)POH-1 and (co)PIOH-2 were shifted towards the upper bound. A position far from the upper bound for the tested and commercial compounds could indicate their relatively low efficiency [49].

## 4. Conclusions

Three new amorphous (co)poly(hydroxyimide)s were synthesized in different molar ratios of ODPA-HAB(D) (3:1, 1:1, and 1:3) with their possible future functionalization. The obtained compounds were characterized by good solubility in common organic solvents (e.g., NMP, DMF, and DMSO), making them easier to process during application.

As a result of the research conducted, the following was shown:

- All of these (co)PIOHs revealed the hydrophobicity. The ODPA content in the molecule mainly influences the water contact angle measure.
- The influence of the stiffening of the polymer chain due to the hindered rotation of the benzene ring with the –CH_3_ groups ((co)PIOH-3) and reduced intermolecular attraction forces in hydrogen bond formation ((co)PIOH-1 and (co)PIOH-2) on the value of T_g_ was observed.
- In the Vis range, with the increase in the HAB contents in the polymer, the less incident radiation was transmitted by the obtained compound.
- The increase in HAB units in copolymer contributed to improving the mechanical properties of the tested materials.
- The gas permeation properties of the tested membranes mainly depend on the intersegment distance and the glass transition temperature of the investigated compounds; thus, the highest permeability coefficients were exhibited by the copolymer with the highest D ratio.

The low permeability of the membranes indicates that the application in systems where such properties are required or the permeation properties of (co)PIOHs can be improved by the previously mentioned modifications, that is, functionalization of the hydroxyl units.

## Figures and Tables

**Table 1:** The characterization of the investigated compounds.

Compound Code	d-Spacing (Å)	Density (g/cm^3^)	*FFV* ^a^	M_n_^b^ (g/mol)	M_w_^c^ (g/mol)	M_w_/M_n_^d^
(co)PIOH-1	5.25	1.3262	0.135	50,000	129,000	2.6
(co)PIOH-2	5.84	1.3115	0.138	34,000	80,000	2.4
(co)PIOH-3	6.43	1.2694	0.142	89,000	209,000	2.3
ODPA-HAB	4.59	1.3657	0.132	65,000	172,000	2.6
ODPA-D	5.33 [49]	1.2531	0.146	23,000	70,000	3.0

^a^ The fractional free volume. ^b^ The number average molar mass. ^c^ The weight average molar mass. ^d^ The dispersity.

**Table 2:** Thermal properties of the examined compounds.

Molecule Code	DSC	TGA		
	T_g_^a^ (°C)	T_5%_^b^ (°C)	T_max_^c^ (°C)	Residual Weight ^d^ (%)
(co)PIOH-1	265	375	407, 521	58
(co)PIOH-2	257	386	410, 520	60
(co)PIOH-3	281	376	432, 508	60
ODPA-HAB	246	405	448, 630	56
ODPA-D	307 [49]	484 [49]	520 [49]	58 [49]

^a^ Glass transition temperature. ^b^ Decomposition temperature of 5% weight loss. ^c^ Temperature of the maximum decomposition rate by DTG. ^d^ Residual weight at 800 °C in nitrogen.

**Table 3:** Mechanical properties of the tested membranes.

Compound Code	E ^a^ (GPa)	R_m_ ^b^ (MPa)	A ^c^ (%)
(co)PIOH-1	3.38 ± 0.14	184.1 ± 11.6	2.7 ± 0.2
(co)PIOH-2	2.76 ± 0.60	167.7 ± 18.0	2.5 ± 0.1
(co)PIOH-3	2.37 ± 0.69	101.1 ± 8.0	2.3 ± 0.1

^a^ Young’s modulus. ^b^ Tensile strength. ^c^ Elongation at break.

**Table 4:** Gas permeabilities and ideal selectivities of the investigated compounds.

Molecule Code	Permeability (Barrer)				Ideal Selectivity	
	N_2_	O_2_	He	CO_2_	α O_2_/N_2_	α CO_2_/N_2_
(co)PIOH-1	0.01	0.09	4.42	0.24	9.00	24.00
(co)PIOH-2	0.06	0.44	9.74	2.29	7.33	38.17
(co)PIOH-3	0.31	1.79	18.37	8.12	5.77	26.19
ODPA-HAB	-	0.01	0.60	0.02	-	-
ODPA-D	1.45 [49]	7.79 [49]	52.40 [49]	31.90 [49]	5.37 [49]	22.00 [49]

The measurements were performed at 30 °C and at 6 bar.

**Table 5:** Gas permeabilities and ideal selectivities of the commercial polyimides [62,63,64,65,66,67].

Molecule Code	Permeability (Barrer)				Ideal Selectivity	
	N_2_	O_2_	He	CO_2_	α O_2_/N_2_	α CO_2_/N_2_
Matrimid^®^_K ^a^	0.32	2.12	-	10.0	6.62	31.25
Ultem^®^_K ^a^	0.052	0.38	-	1.45	7.31	27.88
Kapton^®^_K ^b,c^	0.10 ^b^	0.61 ^c^	8.0 ^b^	2.7 ^b^	6.1	27.0
Kapton^®^_S ^d^	0.049	0.22	-	1.14	4.5	23.27
Kapton^®^_H ^e^	0.0256	0.171	3.20	0.858	6.68	33.52

The measurements were performed at ^a^ 35 °C and at 3.45 bar; ^b^ 35 °C and at 10 bar; ^c^ 35 °C and at 2 bar; ^d^ 35 °C and at 6.8 bar (100 psig); ^e^ 35 °C and at 1.47 bar (1.5 + 0.3 kg/cm^2^).

## Data Availability

The raw data supporting the conclusions of this article will be made available by the authors on request.
